# Quarantine as a public health measure against an emerging infectious disease: syphilis in Zurich at the dawn of the modern era (1496–1585)

**DOI:** 10.3205/dgkh000273

**Published:** 2016-06-06

**Authors:** Gabriella Eva Cristina Gall, Stephan Lautenschlager, Homayoun C. Bagheri

**Affiliations:** 1 Department of Evolutionary Biology and Environmental Studies, University of Zurich, Switzerland; 2 Out-Patient Clinic of Dermatology, Triemli Hospital, Zurich, Switzerland

**Keywords:** early syphilis, quarantaine, Blatternhaus, Renaissance policies, Renaissance epidemiology

## Abstract

Syphilis is considered as one of the most devastating sexually transmitted diseases in human history. Based on historical records, the *“Böse Blattern”* (German for *“Evil Pocks”*) spread through Europe after 1495 and shared symptoms with what we know today as syphilis. Many cities took measures to protect their population. Here, transliterations of archival documents from the 15^th^ and 16^th^ century (provided in the appendix) are used to trace the steps taken by the governing authorities in Zurich to deal with this emerging infectious disease. One of the central measures taken by the city was to establish a quarantine facility referred to as the *“Blatternhaus”.* The city doctors, including the well-known physician and naturalist Conrad Gessner, oversaw the obligatory quarantine and treatment of patients with symptoms. Treatment could range from better nutrition, herbal remedies and skin ointments to aggressive heat therapy and “smoking”. Furthermore, the affliction was suspected as a sexually acquired disease, hence prostitutes and infected foreigners were extradited from the city. Meanwhile, the church used its social influence to promote a more “Christian” behavior. In several respects, the public health measures taken in the 16^th^ century against a new and menacing epidemic do not diverge in their basic rationale from approaches used during the 20^th^ century and today.

## Zusammenfassung

Syphilis gilt als eine der verheerendsten Geschlechtskrankheiten der Geschichte. Historischen Dokumenten zufolge verbreiteten sich die *"Bösen Blattern"*, eine Krankheit mit Symptomen ähnlich der heutigen Syphilis, nach 1495 in Europa und viele Städte ergriffen Maßnahmen, um ihre Bevölkerung zu schützen. Hier nutzten wir Archivmaterial aus dem 15. und 16. Jahrhundert (siehe Appendix) um zu verstehen, welche Schritte die Ratsherren von Zürich einleiteten, um mit dieser neu aufkommenden Krankheit fertig zu werden. Eine der zentralen Maßnahmen der Stadt war die Eröffnung einer Quarantäneeinrichtung, das sogenannte *"Blatternhaus"*. Die Ärzte der Stadt, unter anderem der bekannte Mediziner und Naturforscher Conrad Gessner, überwachten die obligatorische Quarantäne und die Behandlung der Patienten. Die Behandlung reichte von einer besseren Ernährung über pflanzliche Heilmittel und Wundsalben zur aggressiveren Hitzetherapie und dem "Räuchern". Zudem ahnten die Züricher bereits, dass die Krankheit sexuell übertragen wurde und verbannten Prostituierte und Fremde aus der Stadt. Gleichzeitig nutzte die Kirche ihren Einfluss, um mehr "christliches" und gesitteteres Benehmen zu fördern. In vielerlei Hinsicht ähneln die grundsätzlichen Maßnahmen, die im 16. Jahrhundert zum Schutz der öffentlichen Gesundheit gegen eine neue und drohende Epidemie ergriffen wurden, den Ansätzen, die während des 20. Jahrhunderts und heutzutage gebraucht werden.

## Introduction

It is debated whether syphilis was brought to Europe by the American expeditions of Christopher Columbus [1], [2] or whether it was already present in the “Old World” as suggested by microscopic studies of skeletons in England [3]. Molecular phylogenetic approaches [4], [5] have not been conclusive. However, there is less doubt on how and when epidemic syphilis, or the “Great Pox”, started spreading throughout Europe [1], [2], [6]. In 1494 Charles VIII, king of France and presumed legal heir to the reign of Naples, assembled and marched an army of regulars and mercenaries against Naples to claim his inheritance. When the French king and his army returned in 1495, many of his soldiers were ill with “Great Pox”. As the mercenaries were hired from different neighboring countries such as Switzerland [1], there are ample historical records originating from different European cities. 

In the war over Naples, more than 15,000 Swiss mercenaries (called *“Reisläufer”*) were fighting for Charles VIII. The Swiss city of Bern expressed its concern about soldiers with *“Böse Blattern”* coming back from Italy [1]. *Böse Blattern* was the German name used for syphilis from 1496 up to the 17^th^ century. It is sometimes only written as “*Blattern”* or *“Plattern”*, though *Blattern* was previously used for smallpox. The adjective *“Böse”* literally means “evil” or “nasty”. This parallels English usage of the term “Great Pox” to distinguish syphilis from smallpox. Other names for syphilis where: *“Große Blattern”*, *“Franzosen”* and *“Morbus gallicus”*. As syphilis was an unknown disease at the time, and many other infectious diseases were already unsettling the continent, it presented the populace of Europe with a daunting new challenge. 

Syphilis is a sexually transmitted disease (STD) caused by the bacterium *Treponema pallidum* subsp. *Pallidum *[7]. The etiological agent was first isolated by Schaudinn and Hoffman [8] and initially named *Spirochaeta pallida *[9]. The disease is often classified into four stages [10]. Like some other STDs such as AIDS, it can be transmitted from a mother to fetus during pregnancy [11]. In infected women, about 40% of pregnancies lead to stillbirth and neonatal death if infection remains untreated [11]. An infection with syphilis is often linked with a higher risk of HIV infection [12]. Furthermore many infected persons tend to be unaware of infection, as syphilis can be asymptomatic in its early stages. This leads to higher transmission rates to sexual contacts and to children born to infected mothers [11]. 

Since the symptoms of the disease vary with the different stages and are seldom described in detail in older documents, the historical identification of the disease is often based on the names and circumstances given in such documents. When symptoms are described it is often unclear from the original text, whether these are symptoms described by lay people or by doctors, and the language used at the time leaves further room for interpretation [13]. Another problem for the later diagnosis of medieval diseases is the fact that the symptoms of infectious diseases can change over time, often related to a reduction of virulence [13]. Hence, without forensic evidence based on molecular genetics, it is difficult to prove with certainty that the disease known as *Böse Blattern* refers to the syphilis known to us in the 21^st^ century. Nonetheless, it is certain that the history of the social response to syphilis is very tightly knit to the history of the *Böse Blattern* in the German-speaking world. The name “syphilis” appeared for the first time in the 16^th^ century and was used (whether correctly or incorrectly) as a synonym for the* Böse Blattern* or *Franzosen*, and subsequently took over as the name of a disease that shares reported symptoms with the *Böse Blattern*. Therefore, to fully understand the history of syphilis and its impact on society, the study of the *Böse Blattern* and its effect on a medieval society is essential. 

Here we investigated how Zurich, as a semi-autonomous sixteenth century European city reacted to an emerging infectious disease and what measures the city takes today with the numbers of syphilis patients having risen once again. We analyzed primary documents originating in the period between 1496 and 1585 from the city of Zurich, and also reexamined the older secondary literature addressing the *“Böse Blattern”* during the early modern era (Table 1 (Tab. 1)). Furthermore, we briefly look at data from the Swiss *“Bundesamt für Gesundheit”* concerning syphilis, in order to gain perspective on the current situation in Switzerland. The primary documents are from the historical archives of Kanton Zurich.

## Policies of the city upon emergence of the epidemic: quarantine of prostitutes and the ill

**Figure 1:**
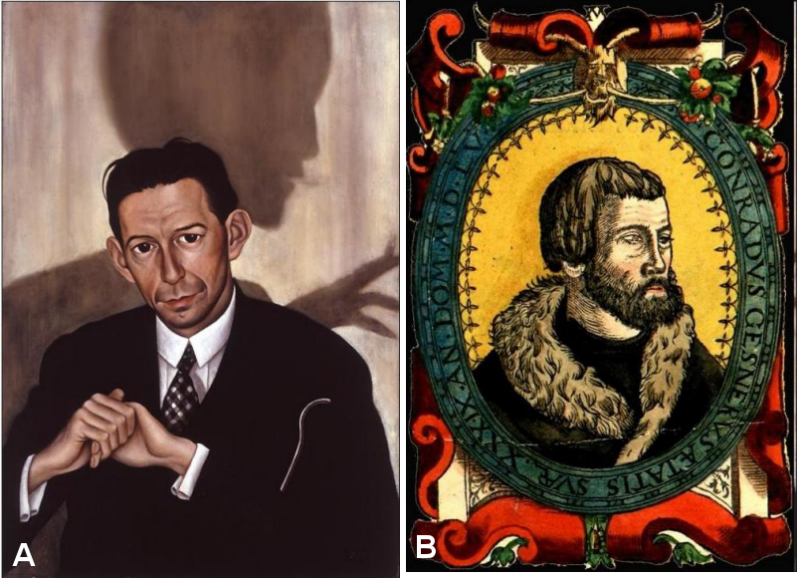
Figure 1A: A 1928 portrait of Hans Haustein (b. 1894) by Christian Schad. Haustein was a leading German dermatologist researching archival documents on the early history of Syphilis. One of his main conclusions was that syphilis arrived on the European public health arena after the discovery of the Americas. He was of Jewish heritage and socialized in Berlin’s left-leaning cultural circles. There are conflicting accounts of his tragic fate after being arrested and harassed by the Gestapo in 1933 [40]. Image of Hans Haustein reproduced with permission from *Museo Thyssen-Bornemisza*, Madrid. B: A woodcut portrait of Conrad Gessner (1516–1565) often printed on the initial pages of his books. Gessner was the principal physician of Zurich and one of the best-known naturalists and physicians in 16^th^ century Europe. He died of the Plague. The year 2016 marks the 500^th^ anniversary of his birth.

**Figure 2:**
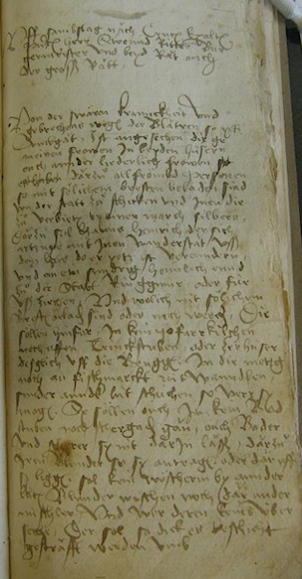
This 1496 Ratsmanual entry (p. 85) is among the first documents with a detailed order of the city council aiming to prevent the further spread of syphilis (here “Blateren”). Photo G. Gall.

Shortly after the first mercenaries arrived with *“Böse Blattern”* in 1496, the city council passed several decrees (Appendix A, B and C ) determining how to handle infected people and stop the disease from spreading. These decrees consist of two *“Abscheide”* and a *“Ratsmanual”* entry. An *Abscheid* is a decree from the Mayor and one or both councils of the city. It deals mostly with supra-regional or “national” affairs. Only parts of the *Abscheide* mentioned here were concerned with the disease. A *Ratsmanual* is a record of decrees from the Mayor and the city councils and mainly addresses local and regional problems. These early documents have more or less the same content concerning the disease. We will focus on two that were already mentioned by the pioneering work of the German dermatologist Hans Haustein [1], which inspired the present study. In the *Abscheid* of 25^th^ May 1496 (see Appendix C ) the council orders that locals with the disease should stay at home and avoid inns and bathhouses. A few months later there is a more detailed decree in the Ratsmanual for the 17^th^ of September 1496 (see Figure 1 (Fig. 1), Figure 2 (Fig. 2) and Appendix A ). The council commanded that all foreigners with *Böse Blattern* were to be sent away and not allowed into the city anymore. In contrast, local people should avoid all parish churches, inns, bathhouses, bridges and markets (specifically the butcher and fish market). Barbers and surgeons were also forbidden to let disease-ridden people into the bathhouses. In addition, washers were forbidden to mix the laundry of sick people with that of healthy people. In this decree, a doctor “Hans Heinrich” is mentioned, though it is not clear what the council orders him to do. All “women of both houses” and other “loose women” (“*liederlich frowen”*) were to be sent away and not to be allowed into the city anymore. The term “loose” as used here is an euphemistic translation of* liederlich*, which is more akin to “skanky”. By “women of both houses” they most likely meant prostitutes belonging to “two houses” that were likely brothels. In Seitz [6] an early order from the 14^th^ century concerning prostitutes is cited, making sure that these women could stay in a special house (probably one of the houses mentioned above) but were not allowed anywhere near crowded places. Since they intended to send away every prostitute (including healthy ones), it appears that prostitutes were seen as a potential transmitter for the disease. This approach was not isolated to Zurich. Jillings [14] cites a similar decree from Aberdeen in 1497, requiring “light” women to stop their “sin of venery” or risk banishment. At least 500 Scottish mercenaries were reportedly in Charles VIII’s army [14]. Wirtz [15] mentions a subsequent decree from 1538 concerning the main brothel of Zurich. In this decree, the city names “ill women” who “deprave” the “fellows” in a way that some of them inherit the* Blattern* and have to live with it for the rest of their lives. Therefore, it is suggested that these women should be sent away and they should look for “clean women”. Other evidence for the idea of prostitutes as transmitters is given by Koebling [16], who cites a text from Felix Platter (1536–1614), a known doctor from Bern. Platter states that according to another authority by the name of Fernel, “a prostitute can however, under certain conditions, transmit the poison of the *Lues* from one man to another without getting sick herself”. It is clear that the authorities were aware of the infectiousness of syphilis as a sexually transmitted disease, though they may have not thought of it exclusively as an STD. They therefore ordered people to stay at home and avoid populated places, as well as indirect infection through clothing. From an epidemiological perspective, this may have been a justified measure given that later dermatological manifestations of syphilis can lead to transmission through non-sexual avenues such as skin contact [17]. 

## Quarantine as public policy: the “Blatternhaus” throughout the 16th century

**Figure 3:**
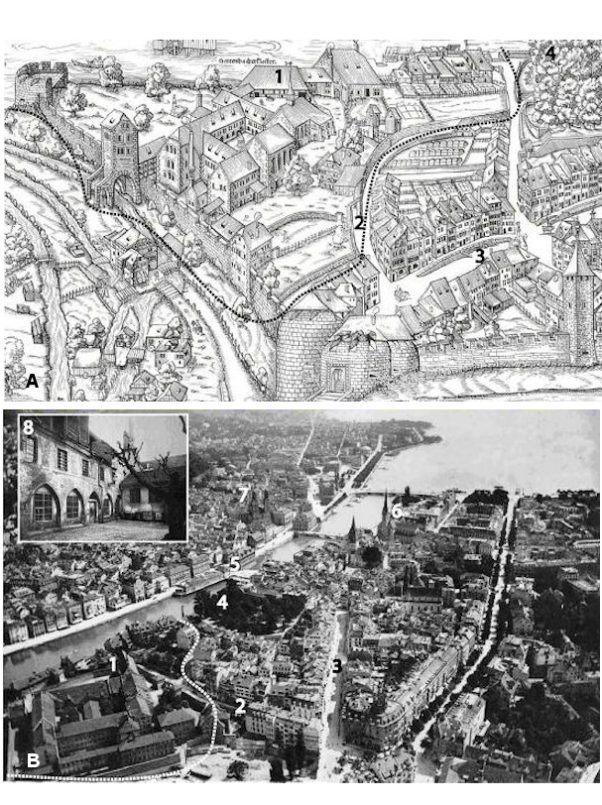
Figure 3A: Partial segment of the *“Murerplan*”, a 1576 woodcut map of Zurich by Jos Murer (1530-1580). The dotted line marks the approximate boundaries of *Kloster Oetenbach*, within which the *Blatternhaus* accomodated and treated syphilis patients. (1) Cloister tower. (2) Future location of Oetenbachgasse (street). (3) Rennweg (street). (4) Lindenhoff (courtyard). B: Aerial photograph of Zurich circa 1898 by Eduard Spelterini (1852–1931). Much of the peripheral cloister structures had been replaced by a city prison built around the older buildings. Dotted lines mark approximate location of previous cloister area. (1) Cloister tower is still present. (2) Oetenbachgasse. (3) Rennweg. (4) Lindenhoff. (5) Rathaus (city hall). (6) Fraumuenster church. (7) Grossmuenster church. (8) Inside view of the cloister courtyard circa 1900, before the structure was destroyed. Cloister tower still visible in background.

Sometime between 1496 and 1525, a specialized quarantine “hospital” named the *“Blatternhaus an Oetenbach”* went into operation. The *Blatternhaus* or *Blatterhus* was a hospital where among others people with *Böse Blattern* were nursed. The *“Archiv-Katalog”* of the *Staatsarchiv Zürich *[18] states that it existed from 1496 to 1842, though we could not corroborate the 1496 starting date from original documents. The first document we found for some kind of official house that was under quarantine was from 1525 (Appendix D ). In the *“Rats und Richtbuch”* from 1525, it is decided that people with the *Blattern* should go to *“Kloster Öttenbach”* (Figure 3A (Fig. 3)), where there would be a place for them to feed and stay. The *Blatternhaus* was hence located inside *Kloster Oetenbach*. This places the *Blatternhaus* between *Oetenbachgasse* (Figure 3B (Fig. 3)) and *Uraniastrasse*, the present location of the *“Urania”* multi-level parking lot in downtown Zurich. Oetenbach was a Dominican cloister for nuns, part of the big Dominican congregation in Zurich. Only nuns and dedicated helpers were allowed to visit and care for the sick. If there was no place in the cloister, then housing would be provided elsewhere. However, the cloister was still expected to provide food for such individuals.

In the *Ratsmanuale*, the first mention of the name *Blatternhaus* is in 1555. In this entry (see Appendix F ), it is ordered that all deficiencies of the *Blatternhaus* should be reported to *“Jacoben”* and *“D. Gessner”* who should than improve the situation. Jacoben is probably Jacob Ruff, a surgeon and friend of Conrad Gessner. Gessner (1516–1565) is one of the most important naturalists and medical doctors in the era, and had an international reputation. He not only described many animal and plant species, but also developed new healing methods based on plants. It is reported that unlike many doctors in the era, he carefully observed patients and applied medicine to their specific needs [19]. He was also the “city-doctor” of Zurich, and had immense influence on the city council and the medical community.

In the *“Blatternhaus-Ordnung”* from 1555, it is stated that because of misuses, no one, neither foreigner nor local, should be let into the *Blatternhaus* unless he/she was first examined by *“Doctor Gessner”* or *“Jacob Ruff”*, or by a *“Meister”*. *Meister* were usually* “Scherer”* or *“Wundärzte”*, the surgeons in that period. Medical men such as Gessner where awarded a doctorate. In contrast, *Scherer* would become *Meister*, a handcraft [20]. If patients had *Plattern* or something similar, they were sent to the *Blatternhaus*. If they had a different disease, they were sent to the* “Spital”* with a note from the examining doctor (Appendix E ). Moreover no one were to be sent anywhere without knowledge of the city council. In the second part of the text, internal affairs are mentioned, as for example the doctors’ pay, *“vier pfund und fünf schilling”*. It is also mentioned that one of the *Scherer* *“Krus”* who seems to have caused some discontent, should stop being lazy and should check his patients regularly. The other four previously mentioned doctors should ensure his dedication and if they realized that he did not work anymore they should bring this to the attention of the council. 

A *Ratsmanual* entry from 1562 (Appendix G ) states that there should be enough to eat and drink, and that someone should look after the sick once or twice a day. The next order found for the *Blatternhaus* was from 1585 and rather detailed and precise in describing the measures taken by the city council (Appendix H ). “Poor sick people” were to be cared for with meat and drink. Many of the same measures as in the order from 1555 are mentioned. Namely, that people should not be taken in by the *Blatternhaus* directly but that they should be examined by a doctor before being sent to the *Blatternhaus*. There, two Doctors were available to examine and treat the sick and to differentiate if they were healed or not. This was stated to be necessary, as some patients did not “reveal” that they were “healing” in order to stay and be fed longer. The text also deals with healing methods. For example, the sick were to be “smoked” and “anointed”, or consumed a potion for perspiration. The doctors had to examine patients in the morning and evening, to make sure that remedies were applied properly, and that patients had enough to eat and drink. The finances and obedience of the patients is also considered. Patients that did not follow doctor’s instructions were to be reported to the city council. If an admonition did not work, they were to be sent away from the *Blatternhaus* regardless of their illness. However, the doctors are mentioned as well, in that there pay was again kept at “vier pfund und fünf schilling” as before and that they should not ask or take more than this amount. Any disregard would be punished. Further, they should not take in additional sick people but should contend with their normal patients. Patients, who were able to pay for themselves, were to buy their own house or be treated at their home. The rest of the text refers to other issues such as the supplies and the servants. 

The church was a leading social institution at the time and took care of the poor, the old and the sick. This explains why the* Blatternhaus* was established in* Kloster Oetenbach*. In addition to food and shelter, it was considered important to attend to people’s psychological needs. *Heinrich Bullinger*, a clerk and friend of *Conrad Gessner*, and the successor of *Zwingli* (a principal figure in the Swiss reformation), gave instructions on how the sick and the dying should be handled [21]. He thought that sick people should devote themselves to the Lord and that everything hard and insufferable was to become easy and comforting as a result. Bullinger stated that the sick should understand that only God could decide whether a person would live or die and that the reason why someone got a disease was up to God alone. Often the “reason” might not be to chasten them, but also to take them away from “this world” and to reward them with eternity. Fortunately, he also thought that it was no sin to turn to a doctor, as the Lord could act through natural and appropriate agents. The doctor used “natural agents” against diseases caused by other natural agents, such as foul air, bad food or stomach disorders. Nevertheless, the church contended that without divine help, no medicine could work. If despite devoting oneself to God the cures did not work, Bullinger recommended that the patient should accept his/her fate, as God had apparently other plans for them [21]. The church saw the main cause for the spread of diseases such as syphilis to be “immoral behavior”. Hence, they pressured the council and the public to attend Mass for moral education [22]. A decree from 1529 promoted by the clergy stated that every healthy citizen should punctually celebrate Mass on Sundays and not leave before it finished [22]. Further, the city council ordered citizens to show more Christian behavior, punishing people who did otherwise [15].

The *Blatternhaus* was an important measure taken by the city against the epidemic and for the intended benefit of the population. As Stein’s authoritative history of the *Franzosenkrankheit* in Augsburg documents in detail, other cities in the German speaking world were building their own quarantine hospitals (some of them also named *Blatternhaus*) [23]. In both the cases of Zurich and Augsburg, the main purpose of the *Blatternhaus* was a place of quarantine for the sick, with the hope of reducing the number of infected in the population. Furthermore, in both cases access to the quarantine facilities was decreed by the city council and regulated by designated medical doctors. The *Blatternhaus* also served as a hospital for healing the infected, where they were nursed and fed. It was seen as a social institution where mainly the poor, who could not afford treatment on their own, where taken up as patients. Therefore, the Zurich council not only tried to avoid the intake of the rich at the *Blatternhaus*, but also tried to avoid patients who only “pretended” to be infected. Doctors where of great importance for the *Blatternhaus*, as they were the ones deciding who would be taken as a patient and decided when a patient was to be released. They were also the ones deciding on the treatment and had to check their patients twice a day to ensure correct treatment. It was also their duty to remedy shortcomings and bring misuses to the attention of the city council. As a result, the city council and the doctors worked hand in hand in the fight against the disease. The church was also of great importance in this relationship, as they provided the staff and the housing for the treatment, and consoled the sick. Furthermore, they exerted influence on the social order in the city along with the city council. 

## The early role of doctors and medical practice in the fight against an emergent epidemic

Doctors played an important role in the fight against diseases. Already in the Zurich decree of 1496 (Appendix A ) it is mentioned that people with *Blattern* should leave the city with a doctor. Ackerknecht [24] mentions that in 1496 the* Heiliggeistspital* (“Holy Spirit Hospital”) engaged a *Blatternarzt* (“*Blattern* doctor”), which indicates that the disease was initially not necessarily cured in the *Blatternhaus*. He also mentions the *Scherer* who were responsible for healing broken bones, and were also trying to cure *“Blattern”*. However, as mentioned by Ackerknecht and the decree from 1496, sick people should not go to the *Scherer’s* practice, and the latter should not allow such visits. Instead, the *Scherer* should medicate them in a quarantine locale or at the patient’s home. 

The city council was well aware of the importance of doctors as a measure to combat disease and heal the sick. Therefore in 1555, the council sent *“Caspar Wolf”* to Montpellier to become a surgeon (*“Wundarzt”*), and *“Georg Keller”* to Padua to become a physician (*“Leibarzt”*) [25] and financed the costs of their studies. Although reports indicate that the virulence of syphilis decreased drastically during the early decades after the first appearance of this disease [26], [27] the measures taken by the governing institutions of Zurich remained mostly the same. In 1552 *Conrad Gessner* published a book in latin on cures and methods of healing [28] *(“Thesaurus Euonymi Philiatri de remediis secretis, liber physicus, medicus et partim etiam chymicus et oeconomicus in vinorum diversi saporis apparatu, medicis et pharmacopolis omnibus praecipue necessarius, nunc primum in lucem editus”*). It cites other doctors, and became one of the most important and used reference books throughout the 16^th^ century. It was translated to German by Hans Nüscher [28] (1583). The book contains several cures concerning the *“Franzosen”* (see Appendix I ). Most involve a mixture of different herbs and some uses of metals such as mercury with salts (*Spießglas*). The medical use of mecury was known from the writings of the Persian physicians Rhazes (865–925 AD) and Avicenna (980–1037 AD), and had been used for skin ailments in the middle ages [29], [30]. Paracelsus promoted its use for syphilis [31]. *Gessner* also developed distilling as a method of producing different drugs. Cures consisted mostly of beverages (*“Wasser”*), ointments and “sweating” or “smoking”. The recipies of* Conrad Gessner* hold an important clue that the disease called *Franzosen* in this text is indeed syphilis. In Appendix I (S. 201) it is mentioned that “the oil is used on the French disease and on the penis” (*“Dises Oel braucht ein fürnemmer Arztet zu den Schwären der frantzosen /und des Mannes glid”*). In the following sentence it is said that the “Geschwär” will be cured with this treatment. The *Geschwär* is probably the “chancre”, one of the symptoms of primary syphilis found in the the genital area. 

## Legacy of 16th century healing methods in the light of subsequent scientific and medical developments

The effectiveness of the cures and the antimicrobial properties of the herbs recommended by Gessner for healing syphilis are unknown. Prior to the discovery of penicillin, treatment of syphilis with soluble salts of mercury remained a mainstay of medical cures up to the nineteenth and early twentieth century [31], [32]. However, it is certain that treatment with mercury had significant toxic side effects [31]. The 16^th^ century methods of “smoking” and “sweating” would have the effect of increasing body temperature. This was echoed in healing methods developed by Wagner von Jauregg in 1918 to heal neuro-syphilis using “fever therapy” [33], [34]. The fever was induced by injecting patients with Malaria-infected blood, subsequently remedied with Quinine. Despite extensive side effects, and a success rate of about 50%, Wagner von Jauregg won the 1927 Nobel prize in Medicine [34].

In considering fever therapy, it is often mentioned in the literature that the bacterium *T. pallidum* is highly sensitive to temperature [7], [35]. Although this is true, our examination of the literature indicates that the statement requires important qualifiers. *T. pallidum* is difficult to grow in culture, hence microbiological experiments are scarce. Fieldsteel et al. [36] were the first to succeed in growing *in vitro* cultures of pathogenically viable *T. pallidum* at 33°C (also Norris [37] at 34°C). They later show [38] that depending on the duration of cultivation (5 to 12 days), the optimum growth temperature is between 33 and 35°C. The data also indicates that at 37°C, bacterial cell counts after 7 to 12 days incubation are significantly lower than those at optimum growth temperatures [38]. However, there is no significant reduction in cell count when comparing 33°C and 37°C growth after 5 days. Hence, the effectiveness of temperature exposure is dependent on the duration of exposure. Meanwhile, results from Stamm et al. [35] show that *T. pallidum* lacks a heat shock response, and after 1.5 hrs incubation at 38°C, there is a markable decrease in protein expression. At 42°C, protein expression is negligible, and the bacteria lose motility. Nonetheless, based on our review of the literature, whether temperatures between 38 and 42°C are primarily bacteriocidal or bacteriostatic is not established. Moreover the clinical studies using malariotherapy where conducted without appropriate control groups [33]. Furthermore, the above studies were conducted once the bacterium had been discovered as a cause for syphilis. Since there is no microbiological evidence that the *“Böse Blattern”*, was caused by *T. pallidum pallidum*, the evaluation of 16^th ^century heat therapy methods is contingent on the assumption that the etiological agents of syphilis and the *Böse Blattern* are the same. 

 In terms of the epidemic control measures at the population level, the quarantine scheme implemented by the city is rather modern in its design. It includes the designation of a facility, the implementation of detection mechanisms, patient care, and patient isolation from the population. In a simulation model of infection dynamics in a quarantine situation, it was found that a city of about 4,000 inhabitants can effectively diminish STD epidemic spread by operating a quarantine facility with a carrying capacity of about 100 patients [39]. This corresponds to the demographics of Zurich in the 16^th^ century. However, it was also found that the key to success in such a scheme is a low rate of false positives and subsequent assignment to the facility [39]. This would depend on the accuracy of symptomatic diagnostics by physicians, or alternatively medical technology in modern times. Indiscriminate assignment of the sick to the quarantine facility generally overflows the system and renders quarantine ineffective. 

## Worldwide resurgence of syphilis in the 21st century: the Swiss case

The discovery of antibiotics in the 1940s revolutionized the treatment of syphilis. Prior treatments such as Malaria therapy or Salvarsan involved complications and potential side effects. Nonetheless, despite the availability of an effective cure, there was a substantial increase in syphilis in Switzerland in the period immediately after World War II that lasted for several years. Incidence rates dropped steadily afterwards, leading to the belief that the disease was conquered. However, along with the liberalization of sexual practices, an unexpected epidemic that mainly affected homosexual men arose in 1962–63 [40]. Between 1973 and 1986, the number of infections reported per year for all sexes averaged above 300 [41]. Subsequently, potentially as a result of changes in sexual behaviour due to the AIDS pandemic, the yearly numbers dropped substantially and remained below 100 for the remainder of the 20^th^ century. AIDS-related mortality in high-risk populations seems to have contributed to this trend [42]. However, there has been a resurgence of syphilis in Europe since the mid 1990’s, and in Switzerland since 2002. This resurgence is problematic. In contrast to HIV transmission, “safer sex” rules focusing on the use of condoms during coitus are inadequate for preventing the transmission of “classic” STIs through unprotected oral sex [43]. Furthermore, given that syphilis had been viewed as “conquered”, public health awareness, symptom recognition and detection was not as effective as desired [43]. In effect, mandatory notification for syphilis in Switzerland was dropped in 1999, only to be reintroduced again in 2006. Since then, according to data of the Federal Office of Public Health (*Bundesamt fuer Gesundheit, BAG*) [41], the number of reported infections per year has risen from 618 in 2006, to 1023 cases in 2011, and stabilized at that level till today (1091 cases in 2015). Unfortunately, until 2007, it was also mandatory to register the full name and address of patients with syphilis and gonorrhoea with the *BAG*. Due to the tendency of physicians to protect their patient’s privacy, these non-anonymized reports may have led to lower return rates and lower quality of reports. 

According to a comparison of European countries by the Robert Koch Institute [44], the per capita rate of syphilis in 2008 placed Switzerland in a leading position in Western Europe. While neighbouring countries such as Germany and Italy reported rates of 3.87 and 2.30 infected per 100,000 inhabitants, respectively, Switzerland reported a rate of 10.03. In 2014 Switzerland reported 12.86 infected per 100,000 inhabitants [41], while the number of infected in Germany nearly doubled to 6.2 per 100,000 inhabitants [45] within only 6 years. A common pattern is a higher incidence rate in males. Between 2011 and 2015, the Swiss incidence rate in males has ranged from 20.3 to 21.6 cases per 100,000 inhabitants, while the corresponding number in females has been between 4.41 and 5.68. The conventional understanding is that a significant proportion of infections occur in men who have sex with men, individuals with many sex partners, and sex workers [41], [46]. Given the non-negligible infection rates in females, one can also expect a resurgence of congenital syphilis. Transmission during pregnancy was reported in 11 cases in 2010 [41]. Given that the subjective course of syphilis is usually asymptomatic, it may now be recommendable to screen for syphilis in pregnancies; especially in cases of unclear exanthemas, patients with other sexually transmitted infections, and those frequently changing sexual partners.

The reason for the relatively high incidence rates in Switzerland in comparison to neighbouring countries is not established. 

## Conclusions: core commonalities of epidemic control 500 years into the modern era

Faced with an epidemic of a new disease, and lacking effective cures, a relatively well organized 16^th^ century city administration resorted to public health management and the handling of infected individuals within a quarantine system. This system included the provision of facilities and personnel, detection of the infected, isolation, and patient care. Furthermore, the city accompanied these measures with public announcements, financial oversight, legal decrees, immigration control, the regulation of sex workers, physician training and experimentation with cures. 

Five hundred years into the modern era, the main advances distinguishing present-day epidemic responses from the 16^th^ century counterpart described here are: better hygiene, more accurate diagnostics, more effective cures, and vaccination. However, as the more recent history of epidemics such as HIV and Ebola have shown, the first line defence against an emergent infectious disease that is reticent to cures, is still based on a combination of the kind of measures implemented by the city of Zurich and similar urban centres in 16^th^ century Europe. 

## Notes

### Competing interests

The authors declare that they have no competing interests.

### Acknowledgement

We thank the *Staatsarchiv* of Kanton Zürich and its staff, where the original documents used in this study were found. Images of old Zurich from Wikimedia commons. This article is dedicated to the admirable contributions of Conrad Gessner and Hans Haustein to our understanding of epidemic disease.

## Supplementary Material

Appendix: Transliterations from original documents

## Figures and Tables

**Table 1:**
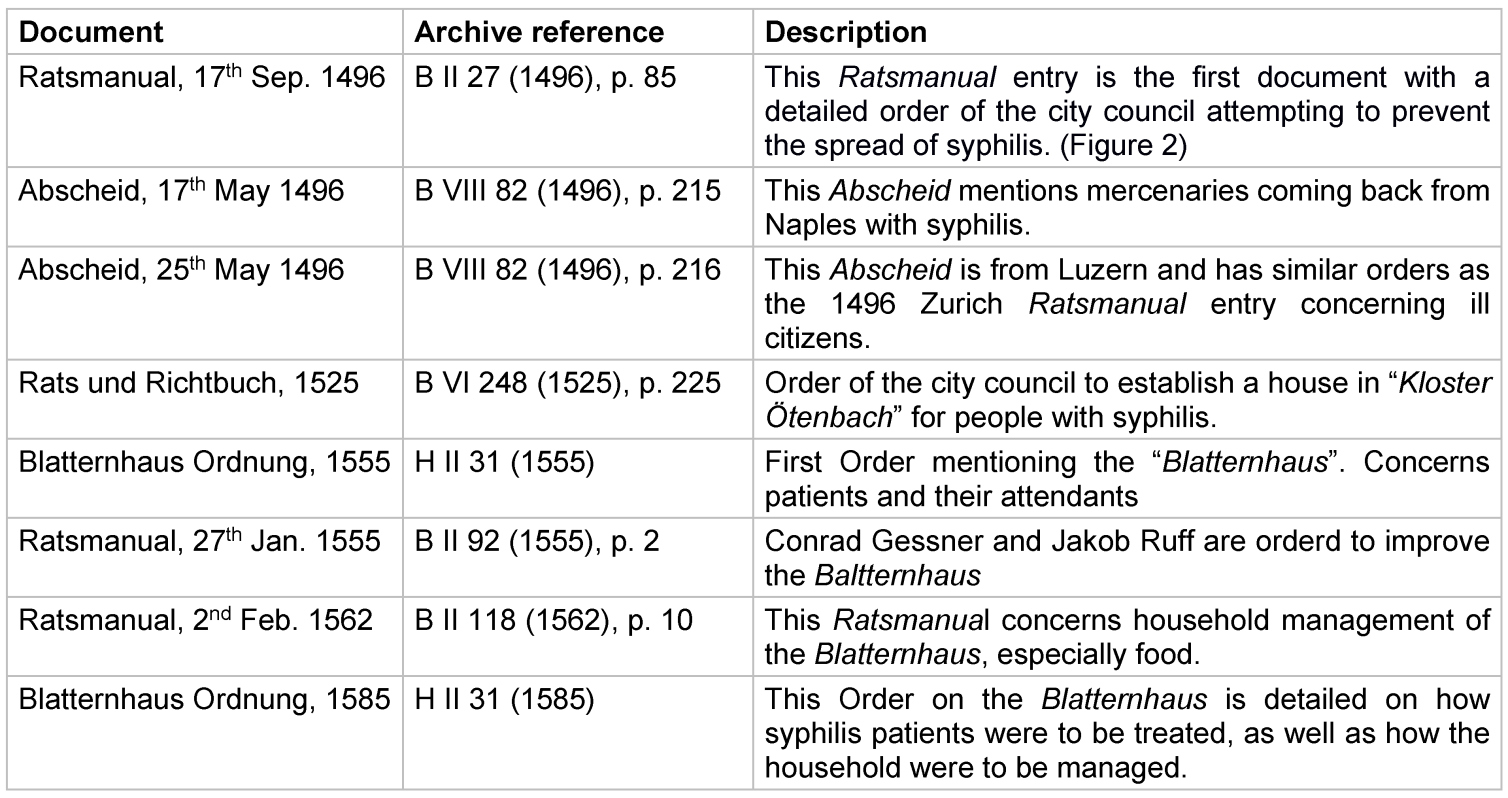
Primary government documents used from the Staatsarchiv Zurich. Transliterations of these texts are provided in the Appendix.

## References

[1] Haustein H (1930). Frühgeschichte der Syphilis: 1495-1498: historisch kritische Untersuchung auf Grund von Archivalien und Staatsdokumenten.

[2] Leven KH (2008). Amerika oder das alte Europa? Der Streit über den Ursprung der Syphilis.

[3] von Hunnius TE, Roberts CA, Boylston A, Saunders SR (2006). Histological identification of syphilis in pre-Columbian England. Am J Phys Anthropol.

[4] Harper KN, Ocampo PS, Steiner BM, George RW, Silverman MS, Bolotin S, Pillay A, Saunders NJ, Armelagos GJ (2008). On the origin of the treponematoses: a phylogenetic approach. PLoS Negl Trop Dis.

[5] Mulligan CJ, Norris SJ, Lukehart SA (2008). Molecular studies in Treponema pallidum evolution: toward clarity?. PLoS Negl Trop Dis.

[6] Seitz FX (1883). Beiträge zur Geschichte der Hygiene und Medicinal-Polizei des Kantons Zürich [Diss].

[7] Lafond RE, Lukehart SA (2006). Biological basis for syphilis. Clin Microbiol Rev.

[8] Schaudinn F, Hoffmann E (1905). Vorläufiger Bericht über das Vorkommen von Spirochaeten in syphilitischen Krankheitsprodukten und bei Papillomen.

[9] Kohl PK, Winzer I (2005). 100 Jahre Entdeckung der Spirochaeta pallida. Hautarzt.

[10] Kent ME, Romanelli F (2008). Reexamining syphilis: an update on epidemiology, clinical manifestations, and management. Ann Pharmacother.

[11] WHO (2007). Information on sexually transmitted infections, Fact sheet N°110.

[12] WHO Europe (2003). Review of Current Evidence and Comparison of Guidelines for Effective Syphilis Treatment in Europe, WHO Europe.

[13] Leven KH, Schlich T (1998). Krankheiten-historische Deutung vs retrospetive Diagnose. Medizingeschichte, Aufgaben, Probleme, Perspektiven.

[14] Jillings K (2010). Plague, pox and the physician in Aberdeen, 1495-1516. J R Coll Physicians Edinb.

[15] Wirtz HG (1909). Grundzüge der öffentlichen Gesundheitspflege im alten Zürich. Festschrift für den Verein für öffentliche Gesundheitspflege.

[16] Koebling HM (1978). Diagnose und Ätiologie der Pest bei Felix Platter (1536-1614), Medizinische Diagnostik in Geschichte und Gegenwart.

[17] Renz-Polster H, Krautzig S, Braun J (2004). Basislehrbuch Innere Medizin.

[18] Archiv-Katalog des Staatsarchiv Zürich. Staatsarchiv Zürich.

[19] Lebert H (1854). Conrad Gessner als Arzt. Akademiesche Vorträge von Züricherischen Dozenten.

[20] Bucher O (1945). Die Anfänge der wissenschaftlichen Anatomie in Zürich für Geschichte der Medizin und Naturwissenschaft. Gesnerus, Schweizerische Gesellschaft.

[21] Campi E, Roth D, Stotz P (2007). Heinrich Bullinger – Schriften.

[22] Brecht E (1985). Wie die Behörden den Zürichern die Lebensfreude verleideten – Kirchenbeschlüsse, Kleiderzucht und Glaubenspflichten von der Reform bis ins 18te Jahrhundert. Tagesanzeiger.

[23] Stein C (2003). Die Behandlung der Franzosenkrankheit am Beispiel Augsburgs.

[24] Ackerknecht E (1972). Medizinische Praxis der Scherer und Bader. Neue Zürcher Zeitung.

[25] Meyer-Ahrens C (1838). Geschichte des Zürischrischen Medizinalwesens.

[26] Knell RJ (2004). Syphilis in renaissance Europe: rapid evolution of an introduced sexually transmitted disease?. Proc Biol Sci.

[27] Tognotti E (2009). The rise and fall of syphilis in Renaissance Europe. J Med Humanit.

[28] Gessner C (1583). Von allerhand kuenstlichen und bewehrten Oelen, Wasseren und heimlichen Artzneyen zu allerley Kranckheiten aussen und in Leib oder sonst zubrauchen; sampt ihrer ordentlichen Bereytung und dienstlichen Figuren. Übersetzt aus dem Lateinischen von H. J. Nüscher.

[29] Lane S (1841). A course of lectures on Syphilis. Lancet.

[30] Proksch JK (1895). Die Geschichte der venerischen Krankheiten. Erster Teil Alterthum und Mittelalter.

[31] O'Shea JG (1990). 'Two minutes with venus, two years with mercury'--mercury as an antisyphilitic chemotherapeutic agent. J R Soc Med.

[32] Brooks M (1910). XIV. The Treatment of Syphilis by Hypodermic Injections of Salicylate of Mercury. Ann Surg.

[33] Kampmeier RH (1980). Wagner von Jauregg and the treatment of general paresis by fever. Sex Transm Dis.

[34] Austin SC, Stolley PD, Lasky T (1992). The history of malariotherapy for neurosyphilis. Modern parallels. JAMA.

[35] Stamm LV, Gherardini FC, Parrish EA, Moomaw CR (1991). Heat shock response of spirochetes. Infect Immun.

[36] Fieldsteel AH, Cox DL, Moeckli RA (1981). Cultivation of virulent Treponema pallidum in tissue culture. Infect Immun.

[37] Norris SJ (1982). In vitro cultivation of Treponema pallidum: independent confirmation. Infect Immun.

[38] Fieldsteel AH, Cox DL, Moeckli RA (1982). Further studies on replication of virulent Treponema pallidum in tissue cultures of Sf1Ep cells. Infect Immun.

[39] Dobay A, Gall GE, Rankin DJ, Bagheri HC (2013). Renaissance model of an epidemic with quarantine. J Theor Biol.

[40] Bohnenblust A (1967). Die Frequenz der Syphilis und der Gonorrhöe an den Schweizerischen Polikliniken für Dermatologie und Venerologie 1917-1966.

[41] Bundesamt für Gesundheit Meldesysteme.

[42] Chesson HW, Dee TS, Aral SO (2003). AIDS mortality may have contributed to the decline in syphilis rates in the United States in the 1990s. Sex Transm Dis.

[43] Lautenschlager S (2005). Sexually transmitted infections in Switzerland: return of the classics. Dermatology (Basel).

[44] Robert Koch Institut (2009). Syphilis in Deutschland im Jahr 2008. Epidemiologisches Bulletin.

[45] Robert Koch Institut (2015). Weiterer starker Anstieg der Syphilis bei MSM in Deutschland im Jahr 2014. Epidemiologisches Bulletin.

[46] Itin P, Bosshard PP, Toutous Trellu L (2015). Syphilis: Diagnostik und Behandlung. Swiss Medical Forum.

