# Supplementary material for: Quarantine as a public health measure against an emerging infectious disease: syphilis in Zurich at the dawn of the modern era (1496–1585)
Source: GMS Hyg Infect Control. 2016 Jun 6;11:Doc13. doi: 10.3205/dgkh000273 (PMC4899769; doi:10.3205/dgkh000273)
Supplement: Appendix: Transliterations from original documents [file HIC-11-13-s-001.pdf]

Quarantine as a public health measure against an emerging infectious disease: syphilis in Zurich at the dawn of the modern era (1496-1585)

Quarantäne als Maßnahme zum Schutz der öffentlichen Gesundheit gegen eine neue Infektionskrankheit: Syphilis in Zürich zu Beginn der Neuzeit (1496-1585)

Gabriella E. C. Gall, Stephan Lautenschlager and Homayoun C. Bagheri

Appendix

Contents:

A) Ratsmanual 17 September 1494, p. 85

B) Abscheid 17 Mai 1496, Stadt Zürich

C) Abscheid 25 Mai 1496, Stadt Zürich

D) Rats und Richtbuch BCI 248, p. 225

E) Ordnung des Blatternhauses 1555

F) Ratsmanual 27 Januar 1555, pp. 1-2

G) Ratsmanual 1562, p. 10

H) Ordnung des Blatternhauses 1585

I) Auszüge aus: Gessner C., *Von allerhand kuenstlichen und bewehrten Oelen, Wassern und heimlichen Artzneyen zu allerley Kranckheiten ausssen und in Leub oder sonst zubrauchen; sampt ihrer ordentlichen Bereytung und diestlichen Figuren*. Übersetzt aus dem Lateinischen von H.J. Nüscher 1583

Attachment 1 to: Gall GE, Lautenschlager S, Bagheri HC. Quarantine as a public health measure against an emerging infectious disease: syphilis in Zurich at the dawn of the modern era (1496–1585). GMS Hyg Infect Control. 2016;11:Doc13. DOI: 10.3205/dgkh000273. Available from: http://www.egms.de/en/journals/dgkh/2016-11/dgkh000273.shtml

Note on transliterations from original documents

With the exception of Appendix A, which (after checking its accuracy) was taken from Haustein's Frühgeschichte der Syphilis: 1495-1498, J. Springer Verlag; 1930, all transliterations were written by G. Gall using original documents from the archives. The occurance of an underscore in brackets (_) indicates the lack of a word, as no transliteration was possible.

A) Ratsmanual 17 September 1494, p. 85

Uff sambstag nach cruces exaltionem herr svennd ritter burgermeister und beyd rät ouch der gross rat beschlossen

Von swären kranckheit und gebrechens wegen der blatren, so yetz umgat, ist angesachen, die gemeinen frowen in beiden hüsern ouch annder liederlich frowen darzu frömbd personen so nit mit söllichen bresten beladen sind, von der statt zu schicken und inen die zu verbieten by einer march silbers. Darzu sol hanns heinrich, der sich artznye mit inen von der stat us dem hus, do er yetz ist, verenndern und an ein sundrig heimlich ennd zu der statt ringgmur oder fürus ziehen. Und welich mit sölichen bresten beladen sind oder noch werden die söllen hinfür in kein pfarrkilchen noch offen trickstuben oder zerhüser desglich uff die bruggen , in die metzg noch an fischmarkt nit wandelnm sunder ander lüt schüchen, so verr sy mögen. Sy söllen ouch in kein badstuben noch schergarden gan, ouch bader und scherer sy nit darin lassen, darzu iren blunder, so sy antragen oder daruff sy liggen, sol kein wöscherin by ander lüten blunder woschen noch dar under mischeln. Und wer deren keins uberseche der sol, so dick es beschicht, gesträfft werden unb

B) Abscheid 17 Mai 1496, Stadt Zürich

Abscheid dess gehaltenen tags uff zinstag our pingsten 1496 (Staatsarchiv Zürich, B VIII 82, p.215)

Absatz 4: Der kriegs knechten halb so jetzmalen mit den bösen blattren beschwerd sind, sol man an bringen unnd darum uff gemelten tag antwurtten damit merer schad unnd gebrest versehen wird

C) Abscheid 25 May 1496, Stadt Zürich

Abscheid zu Luzern uff mitwuch in pfingstfirtagen anno 1496 (Staatsarchiv Zürich B VIII 82, p. 216 recto)

Absatz 2: Uff diesem tag ist angesechen, das jedes ortt mit den sinen, so die bösen blattern habent, verschaffen sol, daheimen in ir huser zu beliben und niena heruss weder zu kilchen, zu stras, in wirtshusern, zu bedern old scherstuben noch an deheim end, da dann die lüt wonung haben, wandlen söllen, als die bott wissent zusagen.

D) Rats und Richtbuch BCI 248 p. 225

Der blaterhyten lüte halb söllent die gemelten (_) in Öttenbach gan, nur den frowen da selbs güttlich oder und besuchtigen, ob man in dem huß uft dem hoft ein stuben und ein blatz finden mächt, die armen lüt inzebeggen und zu qrtznen, und daß man inen uß dem Closter (_) brot und gmüß gebe, biß die selben armen wyter kommen mögen. Uß dem spital sol nun eni (frowe), oder sust anderswo nehmen die söllchen armen lüten pflige.

Und ob sy an ötenbach kein (geshuklig) blatz findent, söllen sy in Sareaments huß und anderstwon süchen, damit man die benempt lüt erhalten myge, zu dem sol man nut desterminder uß ötenbach spyß und trank zinluch geben.
Den so die frowen herüber wyter almüßen geben wöllent, mögen sy Buß uff andern lesthend einer herr oet thün.

E) Ordnung des Blatternhauses 1555

Ordnung wie hinfür die so inn das Blaaterhus und in samlung genommen werden gehalten und des Scheren Belonung sin sol

Als myn Herren von wägen der misbrüchen die in Blaaterhuß an Öttenbach ingeristen wie une wellicher maß die selben abzustellen und zu verbessern nach [niederen] krusten schärer da selbs siner belonung (dirnyl die ander verloren sin soll) zemachen sige , nachtrachtung gehegt. Haben sy daruf zur hütung allerlig gfaaren nachtheil und schadung geordnet und angeschächen namlich das hinfür kheine Personen mer weder frömbd noch heimbsch an Ottenbach (sy komind selbs aber das allemeisten gebe den Lon) noch in den samlung genommen noch empfangen werden. Sy siend dann zuvor von Herrn Doctor Gessner und Jacob Ruff auch Mr. [Lungen] Esslinger und Meister [hrusnstruben]. Als den geordneten zum Blaaterhus ihren gebrästen halb eigentlich besächen und deren dann als solcher besechen wurd und der geschauten personen gepprästen an tag kommen. Ist sie dann mit plattern und desglychen sach behaft soll sy an Ottenbach geordert, ob sy aber mit einer anderen prasten behaftet werre. Als dann zu den Spital gethan und allso de zugefallenen Krankheiten oder ander prästen ordentlich gesundert und underschichen werden. Mit der erlütterung ob schon

die person besächen. Das sy doch weder an Ottenbach noch Spital genommen werden. One miner Herren ((_)Rats) vorwisten und willen damit allerlei gfahren so die [dumen] prästbehaften lut und ihres nutzens und gelegenheit willen bruchen und fürmeiner möchtind, destbast vermitten und erspart blybind,

Und als die Belonung bischar von jeder Person zu Arztnen vier Pfund und fünf Schillling zu Trinlgelt gewesen. Ist genannter meiner Herren Meinung, dass es fürer daby belyben und der krus sich des vergnügen lassen. Doch ob etwa frömd oder frust sonderbar personen verhanden da myn herren allein die spys und sy en lon gebind, sölle er gegen den selben bescheidenlich faren, und niemans wieder überlangen noch beschweren. Wie ihn das vonn mynen herr Bürgermeister lafater. Offtlich wurder sygt und darby verordnet ist sich des wüsten zehalten duch abends und morgens zu den kranken zugend. Und sysnthalb an flys müyg noch Arbeit nit vermindern zerlassen das werde zurwund den kranken zu guten beschliese. Dann wo da nitt beschlossen wrde willicht synthalb wtas anderes gehandelt. Sichergestellt soll durch die abgedachten vier geordneten Herren mit frihe Stellen den scherer zu samlung ernstlich gerecht und vermanet werden, synes unflyses abzestand. Und ob die selben geordneten Herren erkennen möchten das der Krus nit arztnen mer , dann aber in sy ordnung zugijet verdiente. Söllent sie das an ein gesachten Rats bringen wie man sich damit halten wolle. Sie söllen auch Herr Doctor Gessner und Meister Jacob Ruf gewalt haben. Das sy die scherer zu den Kranken bruchent wann es die von nötenbedurft zu besächen und heisten besächen wie dann sy den zerfundwol wusten.

F) Ratsmanual 27 Januar 1555, pp. 1-2

Absatz 2: Die [rechenhanen] sampt den pflegeren in allen sin, sollen an Jacoben und D. Gessner beschicken, über die Mängel des Blatterhuses [raipflagen] und diselb soll möglich zu verbesseren gwalt hab ouch der krusen bestallung der belonung halb, beschern, und den Mangel so er zuvil were, ouch verbesseren, und miz in reden das er bescheidenlig syge,

G) Ratsmanual 1562, p. 10

Montags den 2 februarig, [putz] her müller und beyd räth

Der Schaffene an Öttenbach soll hinfure darzu sechen, das [das] inn den Blatterhus mit äss und thrink [reche] zu [genuge] darinn dhein [unmas] [gedemuchs] und in uns das so darzu gibt glich bschejd und antworte gch und von im ouch die [ger], so mein all vie viel der [unleserlich] ufgeschich wurd, Desglych das alle tag oder das ouch andern tag kin das hus gannge und sache wie [des] darinn [stannte] und hus under [guch] uff den krusen acht habe das [des] alle tag ein mal old zwei [zum] [rechen]genuge,und velliche [unleserlich] sich das die solche abgewyst wird.

H) Ordnung des Blatternhauses 1585

Ordnung und Ansächen über das Blaaterhs an Ötenbach, ouch wie sich die verordneten Herren, desglychen die [unleserlich] , der Amptman der Schärer, der Husknecht und die Kranken halten sollen

Als myn gnedig heren Bürgermeister und Räte der Statt Zürich vor irer gar guter Meinung verordnet und angesächen das arme kranke Lüte gearztet und mit spys und trank erhalten werden söllen und darüber allerlig Ordnung und satzung gemacht, denen aber mit allen [unleserlich] statt beschicht, desglychen ettliche Artikel [unleserlich] von nöten, habend sy nach verhörung des von den verordneten Herren irr Räthen auch ihr Herren [unleserlich] gestalten.

Ratschlag folgende Ordnungen und Gebrüch Stylt und Stett zu halten angesächen, namlich

Das nunhinfür kein Person mer, weder främbd noch heimbsch, sy lonind selbs oder irr Amptman gäbe den Lon an Öttenbach genommen und empfangen werden, sy sygen dann zuvor von Herren Doctor [unleserlich]Reihm und Herr Doctor Caspar Wötten desglychen Meister unter Hans Luch, Meister Heinrich Meyer und Meister Hans Heinrich schüchend. Alls inn geordneten zum Blatterhus oder wer hinfür geordnet werden möchte, allen gemeinlich oder doch inneren theil ihrer geprästen halb eingentlich besächen worden und wen dann söllichs also besächen und der geschauenten Persinen gebprästen an tag kommen also sy mit den Blatteren old anderer desglychen Krankheit behafft, als dann mynen gnädigen Herren anzeigt werden, die mögend sy an Öttenbach [meinen?] und ordern, oder abwysen und mit heuteren geding, kein Person angesagter mynen Herren oder ihr Herren verordneter Vorwissen und bewilligen an Öttenbach genommen werden. Damit allerlig gfahren so die armen Presthaften Luth und ihres nutzes und gliegenheits willen fürwären und bruchen möchten, dester [unleserlich] vermiden und erspart blibing, dann [fuer] der Artztet ein old mer Personen also an [schyn] und [unleserlich-zeichen] arznen wurde man zu kein arzten zugeben verbunden syn.

Und so also derselben Persinen ein oder merern an Öttenbach kommend söllen meister Jacob Buman und ein inder nach in komender arzet dirwyl der selben brästen gar [mingerlig] sind alls etlich allen schlecht offen schäden stlich grosse [unleserlich_tag] in [unleserlich] und allen gliedern etlich linen und anderes haben sler sy all angärtz allenthalben beschauen, die krankheyten eigentlich und gruntlich underschieden damit sy dester so heinung nd ab dem Zaster kommind dann soch oft begibt, das die kranken etwas verschweygend und erst anzeigend wann sy das ersten prästens genässen sind, damit sy lang by spys und trank blyiben könnind, dadurch zweifacher Zaster ufgadt und in nach dem im Schaden ist artznyg und zur darzugegruchen so darzu dient und mit aller kranken one underschid nit einerleig arznyg geheillet und gearzent werden

Als glych zum Anfang inde Person mit rächter darzu dienenden dirunder [unleserlich] [unleserlich], hernach einen Schweiß thrunk gaben, daruf lassen schwitzen und so einer olt ful [sol?] schäden hette sy sygen inn hals oder andersthwo mitsin zu nast flyss wol reinigen, hernach mit räüchen oder salben bis uff syn statt für jaren und sönlcihs alle dag niemanden nach bruchen und kein tag underlassen es beschächen dann [bade] halb

Es sol auch alle Morgen: So lang by inen blyben bis sy alle reäüchekt gesalbt und nach der gepür versorget werden, ouch selbs inen und besonders die räüch und salb ordnen was einen inden zu dinet, damit sy nut u wenig nach [unleserlich] huigend, desglychen sy nerrnach alle nit rechter prdnung schweys verordet werdint.

Und ob etwar ein kranker verstopht were und kin Stulgang hatte das er in denselben fürdere nd sonderlich allen kranken , inmiten der zure damit inen ihr flus, das minder zum hals und mund kömme,

Er soll auch inen allen flyssig zu dem hälfen lugen und so das mul verkompt, sy meistlich zu dem gurgeln mannen mit gurgelwasser, das inen diesntlich syge und den fluss nit zu bals stelle,

Desglychen das er soust alle tag, Abends und Morgens wyllter inen sächen und was von Nöten inen ordnen und nut zu lassen, das sy die kranken einander selbs Räükind , salbind und verbindind (als dann etwan byschächen) sonder er alls der daruf bestellt und besalbet wirt, sönlichs ufzrichten, damit nicht versumpt und vill grosses zapers erspart werde

Und dinwyl dann an ihr zur und arznyg dieser plag trätterlich wie an der spys, ouch ässen und trinlen gebigen ist, sölle irr Arzet sidmalen er am basten wist was einen inder dienet ist, syge zu esen oder zu trinken, vil oder wenig, dem Kächt und der frowen mit ernst [bruchen], das sy den selben, das so er in heyst wund nicht anders, ouch weder minder noch werer gebint

Er der arztet soll ouch so vast er kann, die so er räürkt und salbet, zusammen inn ein stuben liggen, diesälbg fürer heitzen, ouch die kranken nit erst [vegmanen] inn der stuben zu blyben, desglychen irr knächt versächen, das die stuben ouch ander Herren, unden inn huy sympt den fousteren wol beschlossen sygen

Und innsonderheyt sp er der Arzet sich flyssen das er ein indin uffs erhist so muglich ist. Heille und so bald er einer geheillet ist dassälb den verordeten herren kund thun, die dannenthin derselben besächen, ob er heil und gsundsyge oder nut, und wellicher gar gsund ist, denssälben anganz hinweg fertigen, undann irr verordneten herren wüssen gar kiner hinweg gschickt worden, die nach mit heil ewäsen, und so sy dann bals wieder kommen main

mündern Person mit inen haben müssig und sp die verordeten Herren er künnen mächten, das der Arztet in einem old den anderen [fünig] gsyn were, das sy dann des Arzetlos hab, ach gestellt irer sachen gewalt haben inhandten.

Und wellchs under den kranken inen den arzen inn dem so er inen gedient old soust nit gehorsam syn, und das thun [wells], so zur sach dinte, das sölle er ir arzet und der knächt , den verordneten Herren anzeigen, und sy dann mit ernst mit inen reden und zur gehorsame vermanen, und wo eins oder mer, darüber ungehorsam syn, und mynen Herren dasselb anzeigt wirt, dassälb azeigt wellend sy, es syge heil ader nit seyg vertruben und us dem hus verwysen,

Uns alls die belonung bishar von inder Person zartznen vier pfund und fünf schilling zu trink gelt gewäsen, lassen myn herren es fürer dawby belyben, und wellendt ernstlich, das Meister Jacob Buman und ein Inder nochkommender Arzet, sich desselben genztlich vernugen lassen, und von kiner, irr an öttenbach geartzenet wirtm den myn Herren spys und trank und [Gotswillen] gäbend,oder ob glych ener dassälb und irr artzet lon zale nit merer [unleserlich] vordere noch empfange, dann die obgenält bebnung. By gebührender Straf, die myn Herren das iren gwüsslich daruf volgen wurde

Es soll auch mynen Herren gar keine kranken zu (nach wie man spricht, uff den hals Wyssen) sonder sich dere vernügen, so sounst täglich kommend,

Und damit etwan Personen, die den Arzetlon sälbs zergäben und spys und trank zu bezahlen wol vermöchten, inen mynen herren nit uf burden, und also under dem schyn, der armut und synes nutzes an öttenbach kommen, so ist ir mynen Herren ernstlicher will, das erfürderlich ein eigent hus kouffe, dann er sölch Personen erhalten könne, oder inn ein eigen hus artzne,

uff das sy myn Herren , dester minder beschwerd werdint, mit dem heyteren ver[unleserlich] wo er das nit hin thun, das sy myn Herren nach Gedünken haben was sy (sontmalen in das hirver merermaler ouch [bevahlen]) synet halben [fürnynen] und handeln werdint

Und derwyl dann nit wenig ann den gelägen sn wie das die verordneten Herren ouch flyssig zu den kranken luging, ist myner Herren [bruch], das sie beid verordneten Herren, Meister Q. Heinrich Meyer und Meister Hans Heinrich Schücher hinfür ouch mer zu den kranken sächind, sy selbs eigentlich fragind, ob indin geschäche, so da recht syge, ob der Arztet alle tag einmal oder zweig zu inen säche, sy rathsam, und zur heilung düdere oder ob sannst irr artzet oder das wolet, inen das so inen gebürt net verangen lassen, und so sy sämlichs, von indevederen theil erführend, den artzet und das voler warnen und wo sy dannenthin darwieder handeling dassälb gy iren [thrüwen], einen herren Bürgermeister anzeigenndesglychen s y der herren doctoren oder meister Peter Hofnes notthurftig weder du sy die brästen, das sy darzu willig syn söllen

Es söllen ouch der Arzter uff den Knächt desglychen syn frouwen und gsind sodänne der knächt syn frouwen und gsind iff artzet sächen, das er alle tag syn ampt isrichte, und dann herr schaffner uff sy alle sa[m]en, syn flyssige späch und gut sorg haben, das indes dem so es schuldig statt thüge und welliches under inen allen, das ander [farl]esslg findt, dan verordneter herren anzeigen, dieselben dann die schildigen für sich beschicken und mit allen ernst [abzerstan] vermanen, und welliches dann darüber ungehorsam were minen Herren Bürgermeister an zu zeigen, alles by iren [trüwen]

Demnach sol und wirt in Amptmann von kloster Ötenbach uff den Knächt syn frouwen und die kranken, inn das Blaaterhus gäben, allen Gurken, Salz, Gewürz Gemüset, Mäl und was

derglychen von [essiger] spys ist, ouch hloz. Darzu den Knächt und syner Frouwen beiden uff iren byd, alle tag inden zweig drysig brott zweig [querttig] wyn, desglychen an einem Flyschtag sin Pfund Fleisch und an einem Vischtag, zweig bar eiyer und nit mere

Uber die armen kranken, sollent kein [bestimbts] inn esse nach trinken laben, sonders so ir arzet, der dann in syn wirt dem knächt old irr frouwen (wie dann syn ordnung wyst) allengen by syen [thrüw] sägen, nach wys, brotts, flisch, schwach oder stark ist, uff einen tag wol bruchen möge und inen zebruchen gebüre, dassälbig sol dann min amptmann nach notthurft inn das blaaterhus gäben, ouch selbs ein gefliesen ufsächen daruf haben, und der knächt old syn frouw, das alles by dem Amptman reichen, und nyters weder als abstadt gar nit gefordert noch wnommen werden sol

Sodänne sol ein Amptman an Ötenbach inn das vermeilt Blaaterhus gäben, alles [gefider] under und über desglychen häfen [kes] pfannen, und was sounst von [yinnen] [rüpferen] ouch hölzerren gezüg, in ein söllichs [kuch] und hushalt dient und [ufzhar] dahin gedachten knächt, für inn und syn frouw, für ir müyg und Arbeit, alle Jar zwanzig pfund zu richten Jarlon und dru Pfund wäscherlon gäben, und überantworten

Dargägen söllen irr knächt und syn frow die armen kranken früdtlichen und nit trüwen ratsamen, das so dahin gäben wirt geflissenlich kochen inen Betten, wüschen, wäschen, die Notthurftigen lipfern, das holz so zum hus gescheikt wird schyllen, die stuben wermen und sübern, und mit flys versächen

Das die kranken, inn den stuben blybind, ouch sounst die thuren und fänster wol beschlossen sygind velliche kranken etwas erstarrket die von den [badern] inn die ober stuben nämen,

ouch uff das wenigst zur der wuchen alle gmach einst wüschen und indest [asonatz] ein rächte wösch machen, und sounst früyg und spaat alles das thun, was den kranken ir zu guten dienet, und reicht, und ouch die kranken, so dafinden sind, weder inen sälbs, noch zu dem knächt old syner frouwen, weder wüschen noch wäschen. Damit sy sich nit zu klagen habind

Und wenn der Knächt und syn frowe irr bestimmte spys und trank ouch die figer nit bruchen welten noch möchten, söllent sy das gält by dem Amptmann dafür [menyen], und weder das so inen zu stadt noch das so den kranken gehört nit verkouffen, noch im abderwäg us dem hus enthalten, dasälbs noch noturfftig ist versachen und wol hus halten damit nyt veruntruwet werde

Und so der Knächt und syn frowe sy eins old byder obgenalter stucken eins oder merer überträtten [als] were das sy uff die kranken mer nemind , dann sy aber essen noch trinken möchten, old etwas uss den hus verkouffen und hingebind, oder sounst nit den kranken [verträwe] und [unwisch] werind, und inen [m]it telind das die notthurft erfordert oder gastung und ander fersserygen lailten, und dem hus nit flyssig warten
oder von arzet etwas seching das wieder syn ordnung were und dassälb den verordneten Herren nut anzeigen werden, so söllen sy our verzug [gewetanbet] und iren verdienen nach gestraaft. Es sölle ouch vom Amptman hinuf ein flyssgs ufsächen gehept werden, und wo er untrüw old sounst finde das dieser ganzen Ordnung von den arzet, den Knächt, old der Frouwen nit gelebt werde, alls dann mit sampt den pflägern gwalt haben sy zuwarnen und so recht darab getan wurde, angänz zeverlonen, oder irr sachen kinen Herren Bürgermeister anzuzeigen

Und als man vergangens Jars eins üwen Knächts und frouwen bedärffen habendt myn herren [zurathen] [hegel] und Elisabetha [unleserlich] ein jarlang versucheht angenommen, inen diese ordnung verlassen und was von noten mit inen reden lassen, mit der vermanung, das sy sachen und dem allen (iren erwirben nach) flyssig nachkommend. D ann wo das inn eiem old dem anderen nit beschächen, alls dann der amptmann flyssig uffsächen sol, so wurde dementhin der Amptman nach denn in gwalt gäben gegen inen handlen und sy myn Herren ir hand offen haben, in nach dem sy unrächt getan, sy jungfouwe es zurhalten, sounder sy beide alles das ustrichten so von nöten wann sich aber zutrüge das der armen kranken [sanil] werde, das die Notthurft ervordete, inen einen dienst zu zegäben sollent der Amptmann sampt den Pflägern gwalt haben, inen ir etwan ein [unleserlich] minder oder merer Jungfrouwen zuhalten, danit demicht an den armen kranken, nicht versumpt old gespart werde

[Arts] Mittwochs den 18th August Anno 1585 [Pruths] Herr Bürgermeister Thomas und beyd Räth

I) Auszüge aus: Gessner, C., *Von allerhand kuenstlichen und bewehrten Oelen, Wassern und heimlichen Artzneyen zu allerley Kranckheiten ausssen und in Leub oder sonst zubrauchen; sampt ihrer ordentlichen Bereytung und diestlichen Figuren*. Übersetzt aus dem Lateinischen von H.J. Nüscher 1583

Register Angaben zu „Franzosen“: 68.69.179.194.197.201.203.203

Seite 68:

Ein Wasser von vilen stucken zusamen gesetzt / welches sehr wol dienet für die **jenigen so von den bösen Blateren/ welche man franzosen nennet**/ anhrben widerumb auff kommen unnd gesund werden/ wie es der hochgelert unnd verümpte Doctor Rondeletius beschriben hat. Nimm der späne von Blaterholz/ Guaiacum genannt/ mit einem th(r)eheisen gemacht/ i. pfund/ guten alten unverfälschten Triar iiij. Lot/ Conserven von Rosen/ Ochsenzungen/ Boretsch/ jedes iiij. lot. Conserven von Alantwurzel unnd Rosmarin / jedes ii. lot. Des Pulvers elestuarij de gemanis, (ist ein Latwergen von edlem gestein gemacht) unnd letitiae Galeni (ist ein Latwerg/ also genannt/ darumb sie das herz frölich undd leichts muts machen soll/ wirt von denn Arabischen Arzten Galeno zugeschriben) jedes ij. quintle. Solches alles thu inn ein gefäß gefüllt mit gutem weissem Wein/ unnd Wasser/ jeden iij. Quärtle oder vierteil einer Maß/ setze dasselbige in warme äschen/ Nachmals thu Zimmet darzu/ unnd Distilliers: In solchem Wasser zerlaß Zucker/ so vil du bedarffist(seyge es dann durch Wullinen Ermel oder Sack/ durch welchen man pflegt Hyppocras zumachen/ Manica Hyppocratis, hyppocras Ermel genannt (und das ist ein solcher von Leinin oder Wullintuch gemachter Sack/ der under am

boden ganz spitzig ist.) Diß Wasser gibt zurrunden den jenigen/ so von den frantzosen anheben wiederumb auffzukommen.

Seite 68-69:

Theriac Wasser/ des vorgemelten Scribenten. Nimm des alten Theriac ein pfundt/ Sauerampfferen iij. Handvoll/ Camillenblumen/ Poley/Gras/ Cardobendieten/ eines jeden zwo hand voll. Diß alles lasse in gutem weissem firnen Wein wol erbeytzen/ unnd Distilliers darnach mit einander. Das Wasser bahlt zu seinem brauch. Darvon gib einem Krancken wann er zu bett gehen will/ zutrincken iiij. Lot mit vj. lot Sauerampffer unnd Borretsch Wasser gemischet. Diß jetz gemelt Wasser verteibt gwaltigflich die **grossen Schmerzen der franzosen oder bösen Blateren / eintweders** für sich selbs allei getruncken/ oder vermischet mit der Brüy/ dareinn Meerhirsch oder groß Blätern gesoten hat Ich/ spricht Rondelitius, hab glücklich unnd mit grossen lob unnd rum/ vil junge/ altey unnd schwache Personen zu vollkomner heylung gebracht mit disem Wasser/ wänich inen desselbige nun etliche tropffen under dzgmein gesotten tranck vom frantzosen holtz vermischet/zutrincken geben hab. Dann diß tranck tringet schnell hindurch von wegen seiner reinen/ dünnen/ unnd subtilen Substantz/ unnd tribt die frantzosen von innen herauß. Item siß Wasser gemischet under das jenige darinn glüend Gold ist abgelöschen worden/ benimpt dem Quecksilber all sein Schadligleit/ unnd die mengel unnd gebrästen so von dem selbigen sein entstanden/ bringt es widerumb zurecht.

Seite 69:

Ein Theriac wasser/ welches Iacibus Siluius gebracuht hat in der **abscheuliche Kranckheut der frantzosen**. Nimm Gayacholtz ein halb pfund/ Brunnenwasser viij. Pfund/ guten weissen Wein der nut fuß seyge/ ij. pfund/ gebrennt Erdtrauch/ Wegwart/ unnd Camillenwasser/ eines

jeden ein pfund. Solches alles mit einander lasse ein gantze nacht in warmer älschen stehn zubeytzen: Nachmals thu diese nachvolgende stuck darzu: Namlich/ Engelfüß ab den Eychen ein halb pfund/ Epithimi iiij. lot. Spargen rij. Lot. Conserven von Rosen/ Wegwart/Boretsch/ vii Ochsenzungen/ eines heden rij. lot/ guten Theriac/ Alantwurtzel Conferua/ jedes vj. lot. Distillier alles mit einander zugleich/ in einem wol beschloßenen unnd vermachten Doppelem Geschire. Von diesem jetzbeschribenen Wasser gib einem Krancken zutrincken iiij. Oder vj. lot: Wann du willst/ magst wol zu vj. lot des Theriac wassers ij. lot Zucker/ unnd ein quintle Zimmer thun/ vii dann solches widerumb durch hippocras tis Ermel Distillieren lassen/ so wirt es vil ein anmütugern unnd lieblichern gust bekommen. Diß Wasser morgens frü im bett getruncken fürdert und treibt den Schweyß.

Seite 192-194:

Welcher Massen der rote Geist von Spiesglas asgezogen werde/ ein groß Secret oder heimlich verborgen stuck/ eines färnemen Wundartzes. Nimm ij. pfund/ ein und lauter Spießglaß/ zerstosse es zu pulver/ dasselbig thu in ein Irdine Cucurbit/ mach erstlich auff die zwentzig stund lang fein unn ring seuhtlin darunder: Daruff ein sterckers/ und bald noch ein strengers/ biß das sich also sechs und dreisig stund verlossen haben. Oben am Bauch des Pecceptackels/ and derseiten da er ligt/ soltu ein Höltzinen zapffe einstecken/ den selbigen underweilen außziehen/ zuersahten was farb der hinauff gestigen und angeklber geist habe: dann erstlich so ist er weiß/ demnach Wachsgäl/ unnd zum letzten uberal rot. Wann solche Sublimation fertig/ unnd du die Gefäß hast lassen erkalten/ so wüsche das pulver/ so von der hitz des feuscht aus dem Kolben inn des obersten Geschirzs oder Receptackels boden hinauff gestigen ist/ mit einer ferder/ oder einem Hasenfuß sauber herab/ unnd thu es inn ein Retorten/ setze es inn das Marienbad/ unnd laß es also zum anderen mal inn ein gläsin oder steinin Cucurbit

aussdämpffen/ inn welche der Geist oder Dampff vom Spießglas auffriechet oder dampfft/ sollen allweg mit Leininen Tüchlin inn kaltem Wasser genetzt /gefület werden/ dann sie also der Recceptackeln sollen ober sich/ unnd ihr Mundlöcher/ inn welche mann die Häls der Kolben eonstecket/ under sich gesehet sein. Die Kolben /darein das Pulver zusublimiere gethon wirdt/ soll man mit Leym wol verwahren: aber die Recipient kolben/ sollen auß gutem Leymen/ welcher das seuch wl erleiden möge/ gemachet werden. Ihre Häls sollen eines armes lang/ und so weit sein/ daß man mit der Hand samot der Ellenbogen ring aus unnd ein kommen möge. Sie sollen auch ein weiten Bauch haben/ damit sie nicht von den auffsteigenden Geisteren zerspringind/ wie dann hie beygesetzte figur aufweiset.

A. Ist der Ofen/ und das ort da man das feuht einmachen soll.

B. Verzeichnet die Kolben darein das gestossen Spießglas soll gelegt werden.

C. Bedeut den uber Kolbens / durch welchen der Geist oder Dampff des Spießglases in die Recipient gefäß hinauss steiget/ welche auff ein hohes Stüliche gestellt / und besesenet sein/ das sie nicht mögen bewegt werden. Inn den selbigen sihet man ein Nagel oder Zapffen stecken/ welchen so man ihn außziehet/ Kann man sehen wie das Spießglas geserbet seyge/ unnd darnach das feucht sterckeren oder ringeren/ wie es die notturfft erfordert. Von diesem Pulver pflegt obgemelter Wunderartzet nur ein wenig für das Seitenstecjen/Verstopgung der Brust/ unn für die **abscheuliche Krankheut d. franzosen**/ mit bequemen unnd dienlichen Wassern vermischt/ einzugeben.

Etliche sagen für gewüß/ das einer so die **franzosen** hat(möge auff nachfolgende weiß innerthalb derer oder vier tagen geheylet werden: sie stossen den Krancken inn ein Faß/ also das ihme nur allein das Haopt für aus gehet/ Legen ein Eyserinne diecke Platt/ ein wenig glüyend gemacht/ under das durchgelöchert Brett oder Banck/ darauffer sitzen muß/ unnd sprengen auff das selbig das obgenannt Pulver/ also daß ihm der Dampff darvon den Leib under auff pberal berüre/ unnd heissen inne drey stund lang scheitzen/ wönn ers erleiden mag:

wo nicht/ so muß er souil sesto öffter schitzen. Und also werde durch diesen Dampf die Kranckheit genzlich in souil Tagen/ als einer diese Schwitzen gebraucht/ hinweg genommen.

Seite 196-197:

Es schreibt ein Edelman/ welcher das Spießglas wol beryten können/ an Herren Geßner/ er habe sein Spießglas ob den 400. Personen/ Mannen und Weibern/ jungen unnd alten/ nicht nun ob alle gesacgt und nachteil / sonder seht glücklichen: Zu Curinn Pündten hat ers fündd Mans oersonen/ welche das Pestilenzlich fieber gehabt/ eingegebn/ und sie sein widerumb zu ihrer gesundheit komme. Er spricht/ daß es ein gegenwertige und schnelle Arzney für die Pestilenz sey: und denen so man dises eingibt/ beddeffe man weder zuvir/ noch auch darnach ein Ader zuschlage. Sides seines Spießglases hat er herren Geßner etliche Stücklin/ welche dreyerley underschiedene farben gehabt/ zugeschickt/ under den selbigen hielt er das jenige, so rot war/ für das best: darumm das / diesweil es/ damit es ein solche farb gewünne/ lenger müße im feuh: liegen/ sein krafft ausriechete: aber solches müste man auch inn gröfferer dosi eingeben. Einem starcken und wolmögenden/ har obgenannter seines roten Speißglases fünff gran schwer eingegeben: Denen aber so nicht so starck/ und den Weibern/ vier gran. Aber einem blöden und schwachen/ drey gran. Wann aber einer ein sehr starcke Compexion hette/ sagt er/ das man dem selbis gen sicher mdchte sehcs gran/ und nicht darüber eingeben. Wänn es von nöten thüt/ mag man es zu jeder zeit des tags/ ja auch zu Miternacht eingeben/ so sers das man darauff fündd oder sechs stund faste/ unnd zuvor eher das man es einnemme/ man zwi oder drey stund nüchter gewesen sey. Wann es aber nit so not thut/ ist die kommlichste zeit das selbig einzugeben/ morgens früh ein stund voor auffgang der Sonne. Dises Spießglas/ spricht er/ pfleg ich eizugeben für alerley fieber/ wider die Wassersucht/ Gelbsucht/ rot Kur/ oder den Rotenschaden und Blutgang/ Dyssenteria genannt/ für die Melancholy der Weibern/ abfallenden flüß vom haupt/ Hauptwehe/ **Inn sonderheit für die frantzosen**/ harten unnd

verstopfften Bauch/ kalten vollen scheymerigen Magen und Brust/ wider den Szinckenden Athen/ Gifft/ Taubsucht/ unnd andere unsehlich vil Krankheiten. So vil schreibt obgemelter Edelman von seinem Spießglas/

Seite 201:

Das Schwebel Oel/ spricht Leonhartus Fierauquanti im anderen Büch von seinen Caprices/ hat ein solliche fürtreffliche herzliche krafft und tugend/ das niemandts / der sein Effect und würckung nicht gesehen/ dasselbige leichtlich glauben kann. Ich/ sagt er/ habs kein mal nicht gebraucht/ daß ich nicht wunder gespürt und gesehen habe/ innsonderheit/ bezeiget er/ wann es durch den Mund eingegeben wirdt. Man mag aber desselbigen audd vier oder sechs grad schwer/ und nicht darüber/ mit bequemen Wassern/ oder Syrupen ohne sorg eingeben. So mag man es auch wol under allerley Latwerben und Pillulen vermischen/ dann es / auff solche weiß eingegeben/ vil glücklicher würcket. Dienet zu allen Kranckheite/ sie kommend von hitz oder kälte. Es macht auch hüpsch weiß zän/ dann es trocknet hefftig. Dises Oel braucht ein fürnemmer Artzet zu den **schwären der frantzosen**/ und an des Mannes glid. Dan ob es schon anfangs brennet/ so gestillet es doch hernach den schmerzen/ und geheilet das Geschwär fein sauber hinweg. Aber sein brennen wirdt genommen/ wann man ein zerklopfftes Eyerlaar darein thüt(oder mit der Salb aus Bleyweiß gemacht/ oder gewälschenem butter bermischet. Es wirt auch nutzlich gebraucht für das Mundgeschwer der kleinen Kindern für das kalte Feuhz Gangraena gennant/ zu den Wartzen (welche ich an mit selber glücklich erfahren und probiert hab) und für andere schwer heilbare Kranckhheiten

Seite 202-203:

Etliche bereytn es auff ein andere Gattung: Nemlich/ thün sie zu einem theil des gestossne Schwebels/ eben souil Kißlingstein auch zu pulver gestossen in ein Retortglaß/ und

Distillieren bey einnem sanffte Bolfeuhtlin ein sehr güt Oel darauß. Für eas Kranckheiten das selbige gebraucht werde/ worinn(welcher gestalt/ unnd wievil man desselbigen inn einer jeden Kranckheit eingeben sölle/ will ich kurtz anzeigen. Dises Oel werdt nutzlich gebraucht für die kalten Kranckheiten/ so von kalten und faulen feuchtigkeiten/ oder von vil Blästen ihren ursprunng habe: als da sein/ die faulen Dreytägigen/ Viertägigen / unnd allertägliche Fieber: die Pestilenz/Wunden/ hole und löchrige des Hirnes/ des Mundts/ der Zänen/ des Magens/ der Lebern/ des Milzes/ der Müter/ Blaasen/ Därmen/ unnd Gleychen/ so nemlich von uberflüssigen oder faulen feuchtigkeiten herkommen.

Dieses Oel soll mann nun ein wenig mit gebranntem Wasser oder einer Decoction von Breutern/ so nach eines jeden Glides und seines gebrechen qualitet unnd eygenschafft/ bequem und seinstlich seyen/ vermischt/ eingeben/ auff weiß unnd maß wie volget: Ein Vüner federlin soll man inn das Oel stossen/ unnd behend wider und darauß ziegen/ unnd was dann für Oel an dem selbigen begleber/ under das gebränte Wasser/ oder gesochete Brüyen vermischen und dem Krancken eingeben zutrincken. Aber mit welchen Wasseren es sölle inn einer jeden Kranckheit mvermischt werden/ hast du hienach volgend zuvernemmen. Inn dem Alltäglichen Fieber/ ein wenig was seiner ankunfft(soll man es mit Wein/ darinn Roßmarin oder Müntzen gesotten seyge/ zu trincken geben. (…)

Zu dem Siechtag frantzosen genannt/ mit gebranntem Wasser aus Erdrauch und Pfrintmen büet. (..)
